# A Combined CRISP3 and SPINK1 Prognostic Grade in EPS-Urine and Establishment of Models to Predict Prognosis of Patients With Prostate Cancer

**DOI:** 10.3389/fmed.2022.832415

**Published:** 2022-02-17

**Authors:** Lizhu Chen, Enchong Zhang, Johnny Guan, Zhengjie Chen, Jianfeng Ye, Wangmin Liu, Jieqian He, Bo Yin, Yongsheng Song, Mo Zhang

**Affiliations:** ^1^Department of Ultrasound, Shengjing Hospital of China Medical University, Shenyang, China; ^2^Department of Urology, Shengjing Hospital of China Medical University, Shenyang, China; ^3^Department of Urology, David Geffen School of Medicine, University of California, Los Angeles, Los Angeles, CA, United States

**Keywords:** CRISP3, SPINK1, biomarker, EPS-urine, prognostic model

## Abstract

**Background:**

Prostate cancer (PCa) is characterized by significant heterogeneity. Thus, novel prognostic indicators are required to improve prognosis and treatment.

**Methods:**

Cysteine rich secretory protein 3 (CRISP3) and serine peptidase inhibitor Kazal type 1 (SPINK1) levels in expressed prostatic secretion (EPS)-urine collected during digital rectal examination of 496 patients histologically diagnosed with PCa were detected *via* enzyme-linked immunosorbent assay. A combined CRISP3 and SPINK1 prognostic grade (CSPG) was defined using cut-off values from receiver operating characteristic curves. Log-rank Kaplan-Meier survival curves investigated differences in prognosis between groups. Univariate and multivariate Cox analyses investigated the CSPG relationship with biochemical recurrence (BCR), cancer-specific survival (CSS), and overall survival (OS). Three prognostic models were developed and validated.

**Conclusions:**

CRISP3 and SPINK1 levels increased with Gleason score progression, pathological T stage, and metastasis status. CSPG in EPS-urine, which was an effective independent prognostic variable, accurately predicted the prognosis of patients with PCa. Three clinical prognostic models using the CSPG for BCR, CSS, and OS were developed and validated.

## Introduction

As the most common malignancy in males, prostate cancer (PCa) is harmful to men's health (1). According to the latest information from the World Health Organization (https://gco.iarc.fr/), PCa ranks second in the estimated age-standardized incidence rate, first in the 5-year prevalence, and sixth in the age-standardized mortality of all malignant tumors worldwide. At present, clinicians mainly assess the clinical risk level of patients by serum prostate specific antigen (PSA), pathological stage, and Gleason score (2). However, due to the significant heterogeneity of PCa, current clinicopathological prognostic indicators cannot competently predict patient prognosis (3–5). Therefore, novel prognostic indicators are required to improve prognosis and direct prompt treatment.

Using a comprehensive quantitative proteomic analysis, we previously identified and demonstrated that levels of cysteine rich secretory protein 3 (CRISP3) and serine peptidase inhibitor Kazal type 1 (SPINK1) were upregulated in expressed prostatic secretion (EPS)-urine of patients with PCa, compared to those of patients with benign prostatic diseases (6). EPS contains a large number of prostate-secreted proteins and deciduous cells, representing an important source of potential PCa prognostic markers (7). EPS-urine is obtained by collecting the patient's urine after digital rectal examination (DRE), which is more convenient and easier than receiving EPS directly.

Epigenetically regulated by androgen receptor, CRISP3 is upregulated in PCa tissue and higher expression of CRISP3 indicates a worse prognosis (8). Furthermore, low levels of phosphatase and tensin homolog and high CRISP3 effectively predicts biochemical recurrence (BCR) (9). SPINK1 is also associated with PCa; overexpression of SPINK1 is significantly associated with worse cancer-specific survival (CSS) in patients with recurrence after prostatectomy (10). SPINK1 is also a prognostic marker for non-small cell lung cancer and a novel antioxidant promoter during oxidative stress in non-small cell lung cancer (11). Significantly associated with castration-resistant prostate cancer and adverse PSA responses, SPINK1 overexpression serves as a predictor for bone metastases in PCa (12).

To better understand PCa heterogeneity, different types of genetic markers have been proposed in previous studies (3–5). In the present study, we first measured the CRISP3 and SPINK1 expression in the EPS-urine of 496 PCa patients treated with radical prostatectomy. Our results showed that these two proteins in EPS-urine were significant prognosis indicators. Then these two indicators were combined and defined as the combined CRISP3 and SPINK1 prognostic grade (CSPG). Univariate and multivariate Cox analyses indicated CSPG was an effective independent prognostic variable. CSPG and clinicopathologic indicators related to prognosis were also integrated to establish three prognostic models for BCR, CSS and overall survival (OS). Model accuracy was strictly validated in the internal validation group. These findings suggested that CSPG in EPS-urine accurately predicted PCa prognosis.

## Materials and Methods

### Cohort Information

From June 2011 to November 2015, 496 patients histologically diagnosed with PCa from the Department of Urology of Shengjing Hospital of China Medical University were prospectively enrolled into this study. Patients with acute prostatitis or other types of tumors were not included. All patients initially underwent transperineal ultrasound-guided prostate needle biopsy. A mean of 16.4 cores (range: 12–21) were sampled by the template biopsy, based on standardized “Ginsburg protocol” (13). After confirming the diagnosis of PCa, all patients were treated with laparoscopic radical prostatectomy 4 weeks postbiopsy. None of the patients received any kind of therapy, such as adjuvant or neoadjuvant hormonal treatment, radiation therapy, or immune therapy prior to surgical treatment. This research was approved by the Ethics Committee of Shengjing Hospital of China Medical University. Each participant provided signed informed consent.

The clinical and pathological indicators included age, serum PSA, Gleason score, pathological T stage and clinical risk stratification. Each pathological section was reviewed by two urologic pathologists with 10 years of experience, and the Gleason score was recorded based on the original pathology report. Patients were followed-up every 3 months during the first 5 years and then every 6 months. The median follow-up duration was 42.5 months (interquantile range: 29–56 months). Duration of the follow-up was assessed from the date of treatment until the last follow-up or death, which was defined as cancer specific death or a different cause. Metastasis was defined based on lymph node metastasis detected by the pathological report or bone metastasis detected by magnetic resonance imaging and/or radionuclide bone scan. During the follow-up period, a total of 130 patients developed metastatic diseases within a median of 47 (interquartile range: 23–65) months. BCR was defined as PSA ≥ 0.2 ng/ml detected twice after radical prostatectomy. Patients were classified according to the EAU guidelines group risk stratification into low-risk, intermediate-risk, and high-risk levels (2); these indicators are shown in Table 1. To construct and validate effective prognostic models, the patients (*n* = 496) were randomly divided into training (*n* = 377) and validation groups (*n* = 119) *via* “sample” function in R software. The random seeds used in the grouping process were “19970325”.

**Table 1:** The demographics of patients in the study.

**Demographics**	**Value**
Patients(n)	496
Age (median, IQR, years)	63.0 (58.0-71.0)
PSA (median, IQR, ng/ml)	17.25 (12.45-27.10)
Gleason score (n, %)	
≤ 6	133 (26.8%)
7 (34 + )	140 (28.2%)
7 (43 + )	113 (22.8%)
≥ 8	110 (22.2%)
Pathological T stage (n, %)	
pT2	256 (51.6%)
pT3	172 (34.7%)
pT4	68 (13.7%)
Clinical risk stratification (n, %)	
Low	48 (9.7%)
Intermediate	193 (38.9%)
High	255 (51.4%)
CSPG (n, %)	
Grade 1	239 (48.2%)
Grade 2	126 (25.4%)
Grade 3	131 (26.4%)
Metastasis (n, %)	
No	366 (73.8%)
Yes	130 (26.2%)
Surgical margin (n, %)	
Negative	380 (76.6%)
Positive	116 (23.4%)
Biochemical recurrence (n, %)	
No or loss	305 (61.5%)
Yes	191 (38.5%)
Cancer specific survival (n, %)	
No or loss	416 (83.9%)
Cancer specific death	80 (16.1%)
Overall survival (n, %)	
Survival	387 (78.0%)
Death	109 (22.0%)
Follow-up time (median, IQR, months)	42.5 (29.0-56.0)

*IQR, interquartile range*.

### Sample Collection

Serum PSA levels were routinely measured before prostate biopsy. Furthermore, EPS-urine samples were collected for the measurement of CRISP3 and SPINK1 levels, as previously described (6). In brief, three gentle massages were performed on both sides of the median sulcus of the prostate to promote the outflow of prostatic fluid. Then, the patient was instructed to urinate and initial 10 ml of urine containing prostatic fluid was collected. Subsequently, the collected EPS-urine was centrifuged at 14,000 × g for 10 min at 4°C. The supernatant was retained and purified using Amicon Ultra-15 centrifugal filters (3 kDa cutoff; Millipore, Billerica, MA, USA), as instructed in the manual. Finally, 500 μl of purified EPS-urine was collected and stored at −80 °C. The above-mentioned procedures for EPS-urine were in accordance with the Human Kidney and Urine Proteome Project guidelines (14).

### Detection of CRISP3 and SPINK1 in the EPS-Urine *via* Enzyme-Linked Immunosorbent Assay (ELISA)

The EPS-urine protein concentration was quantified by the Bradford method, and equal amounts of EPS-urine protein (100 μg) were loaded into the corresponding wells. Briefly, CRISP3 and SPINK1 levels in EPS-urine were evaluated using ELISA kits (CRISP3: Aviva Systems Biology, OKCD08775; SPINK1: R&D Systems, DY7496-05) according to the manufacturer's instructions. Each urine sample was repeatedly evaluated three times and the mean value was used to reflect CRISP3 or SPINK1 levels in EPS-urine.

### Receiver Operating Characteristic (ROC) Curves

To evaluate the prognostic value of CRISP3 and SPINK1, receiver operating characteristic (ROC) curves of CRISP3 and SPINK1 on CSS were analyzed. The optimal cut-off values of these two variables were obtained to evaluate risk levels of patients from their ROC curves. To validate the accuracy of CSPG and the clinical prognostic models that were constructed, time-dependent receiver operating characteristic curves (tdROC) were utilized. The above procedures were completed using the pROC and timeROC R packages (15).

### Definition of Combined CRISP3 and SPINK1 Prognostic Grade

With the help of GGally R package, it was found that CRISP3 levels were significantly correlated with that of SPINK1; details are given in the results section. Combination of CRISP3 and SPINK1 values were applied to better predict patient risk. Patients were assigned into three subgroups based on the levels of CRISP3 and SPINK1 relative to the optimal cut-off values: grade 1 indicated that CRISP3 and SPINK1 levels were both lower, grade 3 indicated that CRISP3 and SPINK1 levels were both higher, and grade 2 indicated other situations.

### Construction of Clinical Prognostic Models *via* Cox Regression Based on Different Clinical Outcomes

Based on results of multivariate Cox survival analysis, significant variables were used to construct clinical prognostic models on BCR, CSS, and OS in the training group (*n* = 377) *via* survival R package. Then, the regplot in R package was utilized to plot nomograms to visualize the prognostic models. To investigate the accuracy of the three models, tdROC curves were analyzed in the training (*n* = 377) and validation groups (*n* = 119). Then, calibration curves of the three models were plotted *via* nomogramEx in R package. To investigate the roles of CSPG in the three models, C-index values of three clinical prognostic models and clinical prognostic models without CSPG were calculated and displayed by the pec and survival R packages.

### Statistical Analysis

Continuous and categorical variables were expressed as median with interquantile range (IQR) and frequencies with percentages, respectively. Survival analysis was used to evaluate the prognosis of patients with different CSPG grades *via* survival in R package. Survival curves were plotted using survminer in R package, and log-rank test P values were calculated. Clinical outcomes used were BCR, CSS, and OS, separately. Based on the BCR, CSS, and OS, univariate Cox analyses on all clinicopathological variables were performed. BCR, CSS, and OS significant variables were selected separately, and multivariate Cox analyses were also conducted. Differences between groups of continuous variables were investigated using a Wilcoxon test. Differences between groups of discrete variables were investigated using a chi-square test. P < 0.05 was defined as significantly different. Statistical analyses were conducted using R software (version 4.0.4).

## Results

### Comparison of CRISP3 and SPINK1 Expression in PCa With Different Grades and Stages

**Figure 1:**
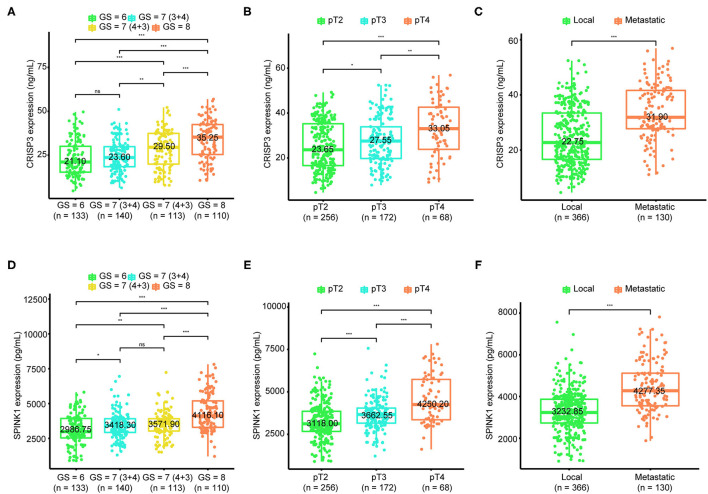
Association of cysteine rich secretory protein 3 (CRISP3) and serine peptidase inhibitor Kazal type 1 (SPINK1) with Gleason score, pathological T stage, and metastasis. The boxplot shows CRISP3 levels increase with the progression of Gleason score **(A)**, the progression of pathological T stage **(B)**, and the progression of metastasis **(C)**. The boxplot plot shows SPINK1 levels increase with the progression of Gleason score **(D)**, the progression of pathological T stage **(E)**, and the progression of metastasis **(F)**. P values are calculated using the Wilcoxon test. ^*^ indicates *P* < 0.05, ^**^ indicates *P* < 0.01, ^***^ indicates *P* < 0.001.

As shown in Figure 1A, the median level of CRISP3 in patients with GS ≥ 8 was 35.25 (25.35–42.45) ng/mL, which was significantly higher than those in patients with GS = 7 (4 + 3) [29.50 (19.90–37.40) ng/mL], GS = 7 (3 + 4) [23.60 (18.40–29.85) ng/mL] and GS ≤ 6 [21.10 (15.33–30.03) ng/mL] (P < 0.01). For SPINK1, the median level was 4,116.10 (3,294.90–5,192.58) pg/mL in patients with GS ≥ 8, and 3,571.90 (3,031.50–3,915.30) pg/mL in patients with GS = 7 (4 + 3), and 3,418.30 (2,930.90–3,912.75) pg/mL in patients with GS = 7 (3 + 4), and 2,986.75 (2,536.65–3,932.98) pg/mL in patients with GS ≤ 6 (*P* < 0.01, Figure 1D). Increased with the progression of pathological T stage, the median level of CRISP3 in patients at pT2 was 23.65 (16.60–35.25) ng/mL, lower than patients at pT3 [27.55 (19.75–33.90) ng/mL] and pT4 [33.05 (23.85–42.63) ng/mL] (*P* < 0.05, Figure 1B). As shown in Figure 1E, the expression of SPINK1 increased with the progression of pathological T stage [pT2: 3118.00 (2,666.40–3,856.43) pg/mL; pT3: 3,662.55 (3,153.33–4,045.35) pg/mL; pT4: 4,250.20 (3,354.83–5,730.93) pg/mL; *P* < 0.001). CRISP3 and SPINK1 levels were higher in 130 metastatic patients (Figures 1C,F, *P* < 0.0001). The median expression of CRISP3 in metastatic PCa patients was 31.90 (27.73–41.65) ng/mL, while the median level of CRISP3 in non-metastatic patients was 22.75 (16.63–33.45) ng/mL. The median level of SPINK1 in metastatic patients was 4,277.35 (3,554.45–5,117.23) pg/mL, which was significantly higher than those of non-metastatic patients [3,232.85 (2729.18–3,868.40) pg/mL]. Taken together, our findings indicated that CRISP3 and SPINK1 expression was positively associated with PCa progression.

### CRISP3 and SPINK1 Expression Was Significantly Correlated With Aggressive PCa Clinicopathological Characteristics

**Figure 2:**
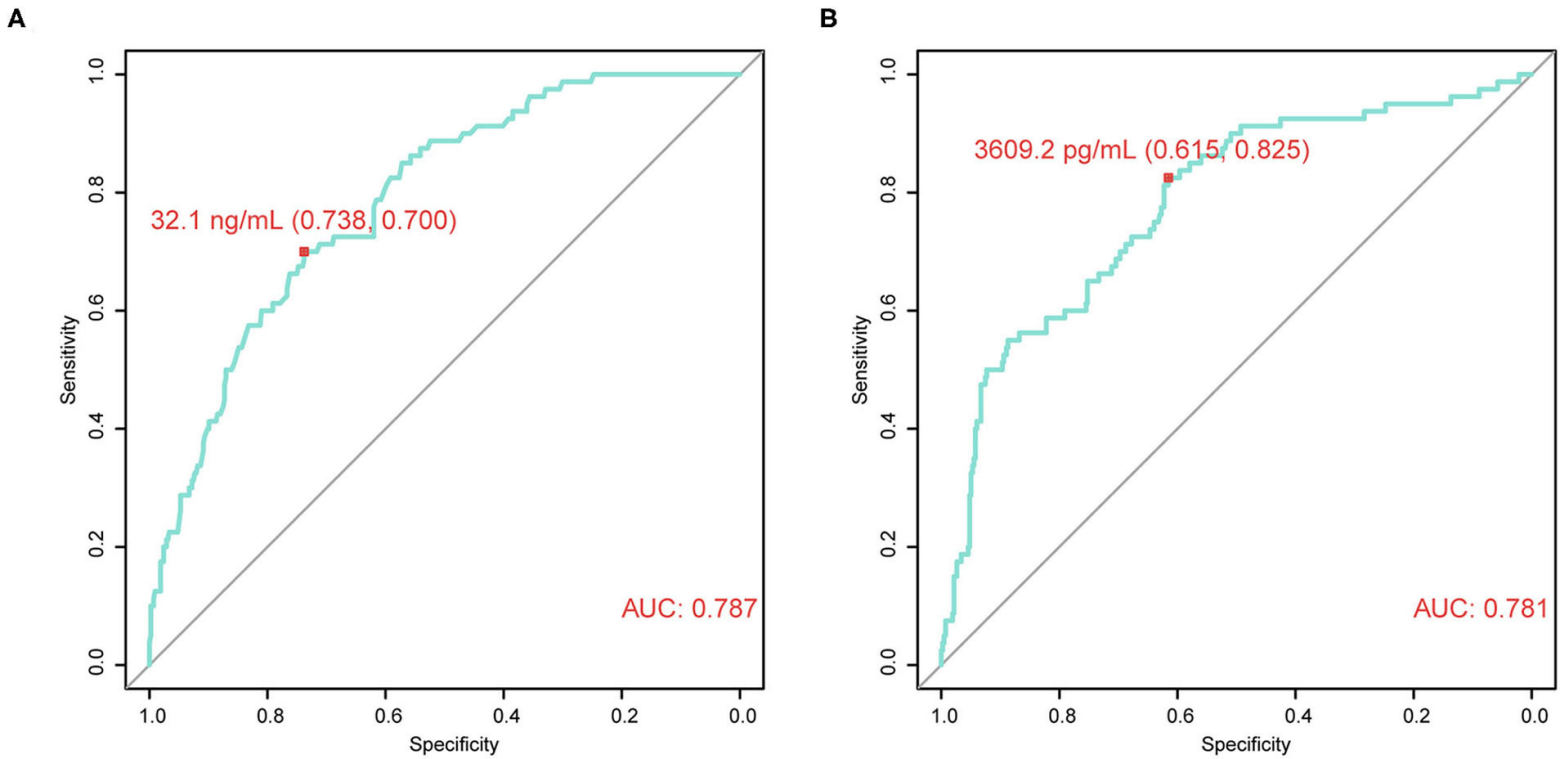
Time-dependent receiver operating characteristic (tdROC) curves reveal good predictive effect and determine optimal cut-off values of cysteine rich secretory protein 3 (CRISP3) and serine peptidase inhibitor Kazal type 1 (SPINK1). **(A)** CRISP3 tdROC curve indicates that the optimal cut-off value is 32.1 ng/ml (sensitivity: 73.8%; specificity: 70.0%). **(B)** SPINK1 tdROC curve indicates that the optimal cut-off value is 3609.2 pg/ml (sensitivity: 61.5%; specificity: 82.5%).

Next, the association between CRISP3 and SPINK1 expression and clinicopathological characteristics of patients with PCa was explored. According to the ROC curve for survival analysis (CSS), the optimal cut-off value of CRISP3 was 32.1 ng/ml (sensitivity: 73.8%; specificity: 70.0%), with the area under curve (AUC) of the ROC curve 0.787; and the optimal cut-off value of SPINK1 was 3609.2 pg/ml (sensitivity: 61.5%; specificity: 82.5%), with the AUC 0.781 (Figures 2A,B). Based on CRISP3 and SPINK1 optimal cut-off values, all patients were divided into a high (*n* = 330, 66.53%) or low CRISP3 group (*n* = 166, 33.47%), and a high (*n* = 270, 54.44%) or low SPINK1 group (*n* = 226, 45.56%). These data demonstrated that there was a significant correlation between high CRISP3 expression and aggressive clinicopathological characteristics, including positive surgical margin (*P* = 0.022), high Gleason score (*P* < 0.001), advanced pathological T stage (*P* = 0.001), and metastasis (*P* < 0.001). Meanwhile, there were significant differences in serum PSA (*P* = 0.002), surgical margin (*P* < 0.001), Gleason score (*P* < 0.001), pathological T stage (*P* = 0.001), metastasis (*P* < 0.001), and clinical risk stratification (*P* < 0.001) between the high and low SPINK1 groups. Other details are shown in Table 2.

**Table 2:** The association of CRISP3 and SPINK1 with clinical information of patients.

	**CRISP3**			**SPINK1**		
	**Negative (*n =* 330)**	**Positive (*n =* 116)**	***P*** **value**	**Negative (*n =* 270)**	**Positive (*n =* 226)**	***P*** **value**
Age	63.0 (59.0-71.0)	63.0 (58.0-71.0)	0.430	63.0 (58.0-71.0)	63.0 (58.0-71.2)	0.882
PSA	17.3 (12.35-27.55)	18.75 (12.50-26.38)	0.772	15.2 (11.18-26.65)	19.6 (13.63-27.55)	0.002
Surgical margin			0.022			<0.001
Negative	263	117		235	145	
Positive	67	49		35	81	
Gleason score			<0.001			<0.001
≤ 6	106	27		91	42	
7 (34 + )	111	29		96	44	
7 (43 + )	66	47		46	67	
≥ 8	47	63		37	73	
Pathological T stage			0.001			<0.001
pT2	175	81		170	86	
pT3	123	49		77	95	
pT4	32	36		23	45	
Metastasis			<0.001			<0.001
No	264	102		233	133	
Yes	66	64		37	93	
Clinical risk stratification			0.120			<0.001
Low	33	15		32	16	
Intermediate	138	55		123	70	
High	159	96		115	140	

*Differences between groups of continuous variables were investigated by Wilcoxon test. Differences between groups of discrete variables were investigated by chi-square test*.

### CSPG Can Be Used to Predict Prognosis of Patients With PCa

**Figure 3:**
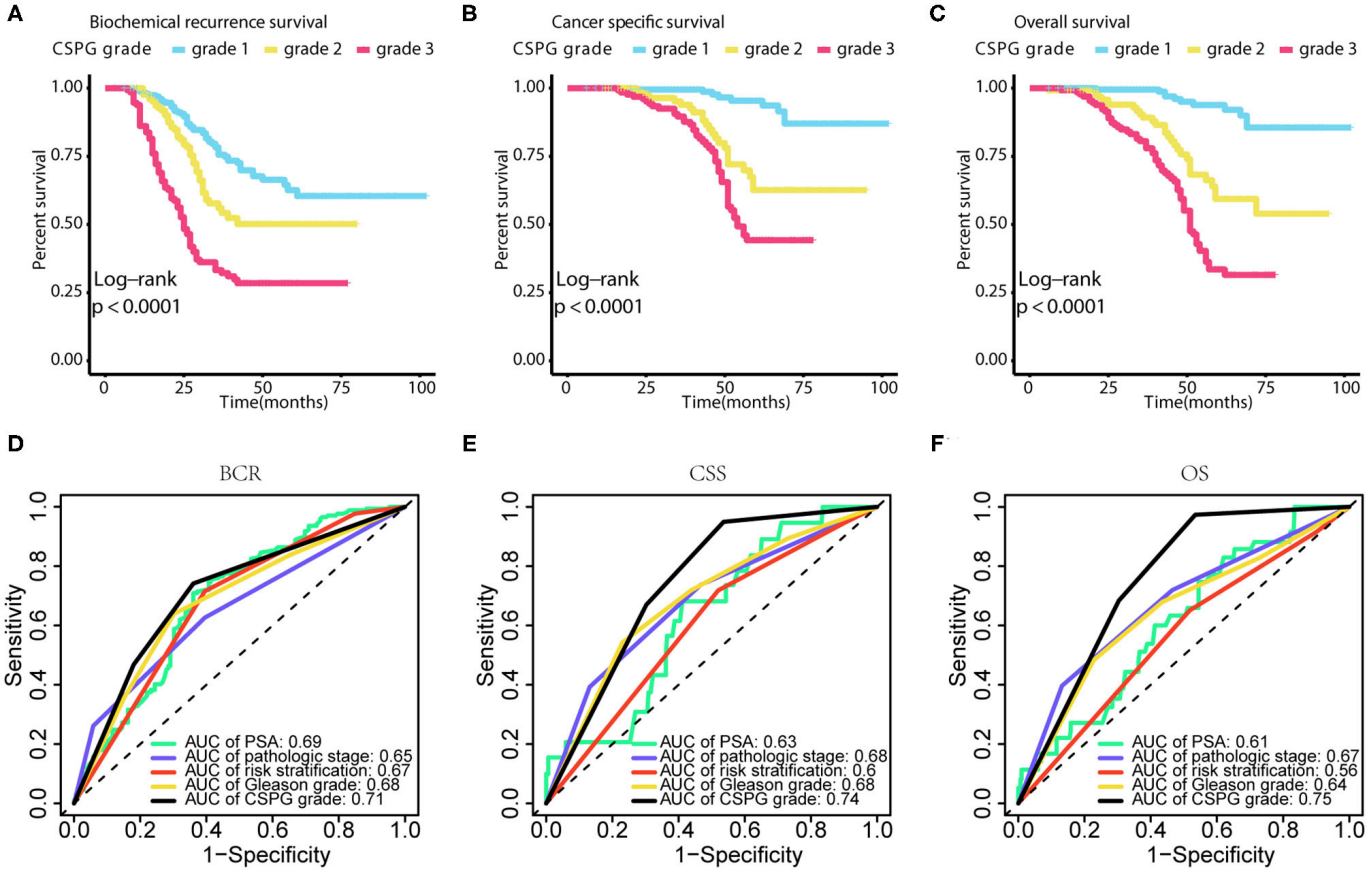
Survival curves and tdROCs of combined cysteine rich secretory protein 3 (CRISP3) and serine peptidase inhibitor Kazal type 1 (SPINK1) prognostic grade (CSPG) based on different survival events. These survival curves reveal that BCR [**(A)**, log-rank test *P* < 0.0001], CSS [**(B)**, log-rank test P < 0.0001], and OS [**(C)**, log-rank test P < 0.0001] worsen with increased CSPG grade. And these tdROCs reveal that the accuracy of CSPG was highest in 3-year tdROC compared with others clinical parameters for BCR [**(D)**, AUC = 0.71], CSS [**(E)**, AUC = 0.74], and OS [**(F)**, AUC = 0.75]. TdROC, time-dependent receiver operating characteristic curve; BCR, biochemical recurrence; CSS, cancer-specific survival; OS, overall survival.

**Figure 4:**
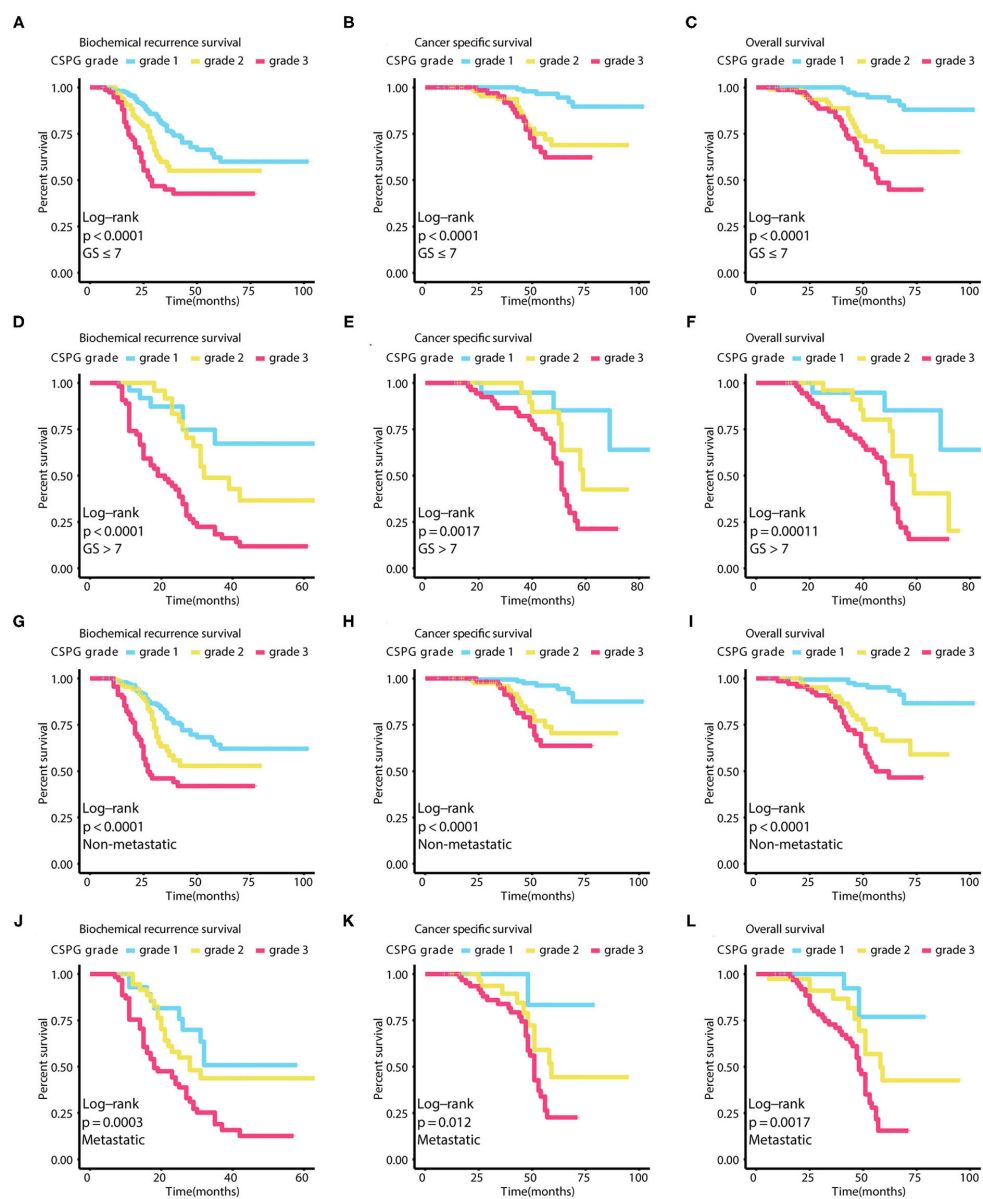
Survival curves in different subgroups of combined cysteine rich secretory protein 3 (CRISP3) and serine peptidase inhibitor Kazal type 1 (SPINK1) prognostic grade (CSPG) based on different survival events. These curves reveal that BCR worsens with increased CSPG grade in patients whose Gleason score ≤ 7 [**(A)**, log-rank test P < 0.0001], in patients whose Gleason score > 7 [**(D)**, log-rank test P < 0.0001], in patients without metastasis [**(G)**, log-rank test P < 0.0001], and in patients with metastasis [**(J)**, log-rank test P = 0.0003]. These curves reveal that CSS worsens with increased CSPG grade in patients whose Gleason score ≤ 7 [**(B)**, log-rank test P < 0.0001], in patients whose Gleason score > 7 [**(E)**, log-rank test P = 0.0017], in patients without metastasis [**(H)**, log-rank test P < 0.0001], and in patients with metastasis [**(K)**, log-rank test P = 0.012]. These curves reveal that OS worsens with increased CSPG grade in patients whose Gleason score ≤ 7 [**(C)**, log-rank test P < 0.0001], in patients whose Gleason score > 7 [**(F)**, log-rank test P = 0.00011], in patients without metastasis [**(I)**, log-rank test P < 0.0001], and in patients with metastasis [**(L)**, log-rank test P = 0.0017]. BCR, biochemical recurrence; CSS, cancer-specific survival; OS, overall survival.

Using GGally in R package, we found CRISP3 and SPINK1 levels were significantly correlated with Pearson correlation coefficient = 0.629 in all patients, and Pearson correlation coefficient = 0.771 in low-risk patients, and Pearson correlation coefficient = 0.653 in intermediate-risk patients, and Pearson correlation coefficient = 0.569 in high-risk patients (*P* < 0.001) (Supplementary Figure 1). As shown in Figures 3A–C, PCa prognosis worsens with an increased CSPG grade (BCR: log-rank *P* < 0.0001; CSS: log-rank *P* < 0.0001; OS: log-rank *P* < 0.0001). And the accuracy of CSPG was highest in 3-year tdROC, compared with others clinical parameters today used in clinical practice (Figures 3D–F). Furthermore, in the subgroup analysis of patients with PCa with a Gleason score ≤ 7 (Figures 4A–C), prognosis worsened with increased CSPG grade (BCR: log-rank *P* < 0.0001; CSS: log-rank *P* < 0.0001; and OS: log-rank *P* < 0.0001). Similarly, in patients whose Gleason score > 7 (Figures 4D–F), prognosis worsened with increased CSPG grade (BCR: log-rank *P* < 0.0001; CSS: log-rank *P* = 0.0017; and OS: log-rank *P* = 0.00011). In addition, CSPG was significantly positively associated with poor prognosis in patients with (BCR: log-rank *P* < 0.0001; CSS: log-rank *P* = 0.0017; and OS: log-rank *P* = 0.00011) or without metastases (BCR: log-rank *P* = 0.0003; CSS: log-rank *P* = 0.012; and OS: log-rank *P* = 0.0017) (Figures 4G–L). Taken together, these findings suggested that CSPG grade had a good predictive effect on PCa prognosis.

### Univariate and Multivariate Cox Analyses Confirmed CSPG Is an Effective Independent Prognostic Variable

Univariate Cox analyses indicated that Gleason score, pathological T stage, metastasis, clinical risk stratification, and CSPG were significant predictors for BCR, CSS, and OS. Serum PSA was correlated with BCR, but not with CSS or OS. These variables were selected to further perform multivariate Cox analyses; PSA (HR = 1.016, 95% CI: 1.005–1.026, *P* = 0.004), metastasis (HR = 1.016, 95% CI: 1.005–1.026, *P* = 0.004), and CSPG (Grade 2: HR = 1.364, 95% CI = 0.920–2.023, *P* = 0.122; Grade 3: HR = 3.140, 95% CI = 2.177–4.528, *P* < 0.001) were independent predictors for BCR. For CSS, Gleason score (HR = 1.902, 95% CI: 1.060–3.412, *P* = 0.031), pathological T stage (HR = 1.748, 95% CI: 1.069–2.857, *P* = 0.026), metastasis (HR = 1.816, 95% CI: 1.116–2.955, *P* = 0.016), and CSPG (Grade 2: HR = 3.456, 95% CI = 1.565–7.672, *P* = 0.002; Grade 3: HR = 5.718, 95% CI = 2.685–12.177, *P* < 0.001) were independent predictors. For OS, Gleason score (HR = 1.773, 95% CI: 1.065–2.950, *P* = 0.028), pathological T stage (HR = 1.663, 95% CI: 1.099–2.517, *P* = 0.016), metastasis (HR = 1.629, 95% CI: 1.077–2.463, *P* = 0.021), and CSPG (Grade 2: HR = 3.942, 95% CI = 1.934–8.032, *P* < 0.001; Grade 3: HR = 7.481, 95% CI = 3.829–14.614, *P* < 0.001) were independent predictors. All univariate and multivariate Cox analyses details are displayed in Tables 3, 4. The above findings confirmed that CSPG was an effective independent prognostic variable for BCR, CSS, and OS.

**Table 3:** Results of univariate Cox analysis.

	**BCR**		**CSS**		**OS**	
	**HR**	***P*** **value**	**HR**	***P*** **value**	**HR**	***P*** **value**
Age	0.99 (0.98-1.02)	0.915	1.02 (0.99-1.05)	0.141	1.01 (0.99-1.04)	0.228
PSA	1.03 (1.02-1.03)	<0.001	1.01 (1.00-1.03)	0.057	1.01 (0.99-1.02)	0.065
Gleason score						
≤ 7	1		1		1	
> 7	2.493 (1.846-3.366)	<0.001	3.780 (2.431-5.828)	<0.001	3.326 (2.279-4.855)	<0.001
Pathological T stage						
pT2	1		1		1	
pT3/pT4	1.873 (1.403-2.502)	<0.001	2.721 (1.695-4.368)	<0.001	2.479 (1.662-3.696)	<0.001
Metastasis						
No	1		1		1	
Yes	3.106 (2.319-4.160)	<0.001	4.185 (2.682-6.528)	<0.001	3.669 (2.508-5.368)	<0.001
Clinical risk stratification						
Low/intermediate	1		1		1	
High	2.616 (1.922-3.562)	<0.001	2.246 (1.392-3.625)	0.001	1.871 (1.258-2.782)	0.002
CSPG						
Grade 1	1		1		1	
Grade 2	1.817 (1.238-2.668)	<0.001	9,619 (4.704-19.619)	<0.001	11.376 (6.003-21.556)	<0.001
Grade 3	4.050 (2.894-5.668)	<0.001	2.246 (1.392-3.625)	0.001	1.871 (1.258-2.782)	0.002

*BCR, biochemical recurrence; CSS, cancer-specific survival; OS, overall survival*.

**Table 4:** Results of multivariate Cox analysis.

	**BCR**		**CSS**		**OS**	
	**HR**	***P*** **value**	**HR**	***P*** **value**	**HR**	***P*** **value**
PSA	1.016 (1.005-1.026)	0.004	NA	NA	NA	NA
Gleason score						
≤ 7	1		1		1	
> 7	1.089 (0.724-1.639)	0.682	1.902 (1.060-3.412)	0.031	1.773 (1.065-2.950)	0.028
Pathological T stage						
pT2	1		1		1	
pT3/pT4	1.269 (0.935-1.722)	0.127	1.748 (1.069-2.857)	0.026	1.663 (1.099-2.517)	0.016
Metastasis						
No	1		1		1	
Yes	1.830 (1.327-2.525)	<0.001	1.816 (1.116-2.955)	0.016	1.629 (1.077-2.463)	0.021
Clinical risk stratification						
Low/intermediate	1		1		1	
High	1.556 (0.990-2.445)	0.055	0.993 (0.537-1.836)	0.983	0.884 (0.527-1.482)	0.640
CSPG						
Grade 1	1		1		1	
Grade 2	1.364 (0.920-2.023)	0.122	3.465 (1.565-7.672)	0.002	3.942 (1.934-8.032)	<0.001
Grade 3	3.140 (2.177-4.528)	<0.001	5.718 (2.685-12.177)	<0.001	7.481 (3.829-14.614)	<0.001

*BCR, biochemical recurrence; CSS, cancer-specific survival; OS, overall survival*.

### Three Clinical Prognostic Models Using CSPG Were Developed and Validated for BCR, CSS, and OS

**Figure 5:**
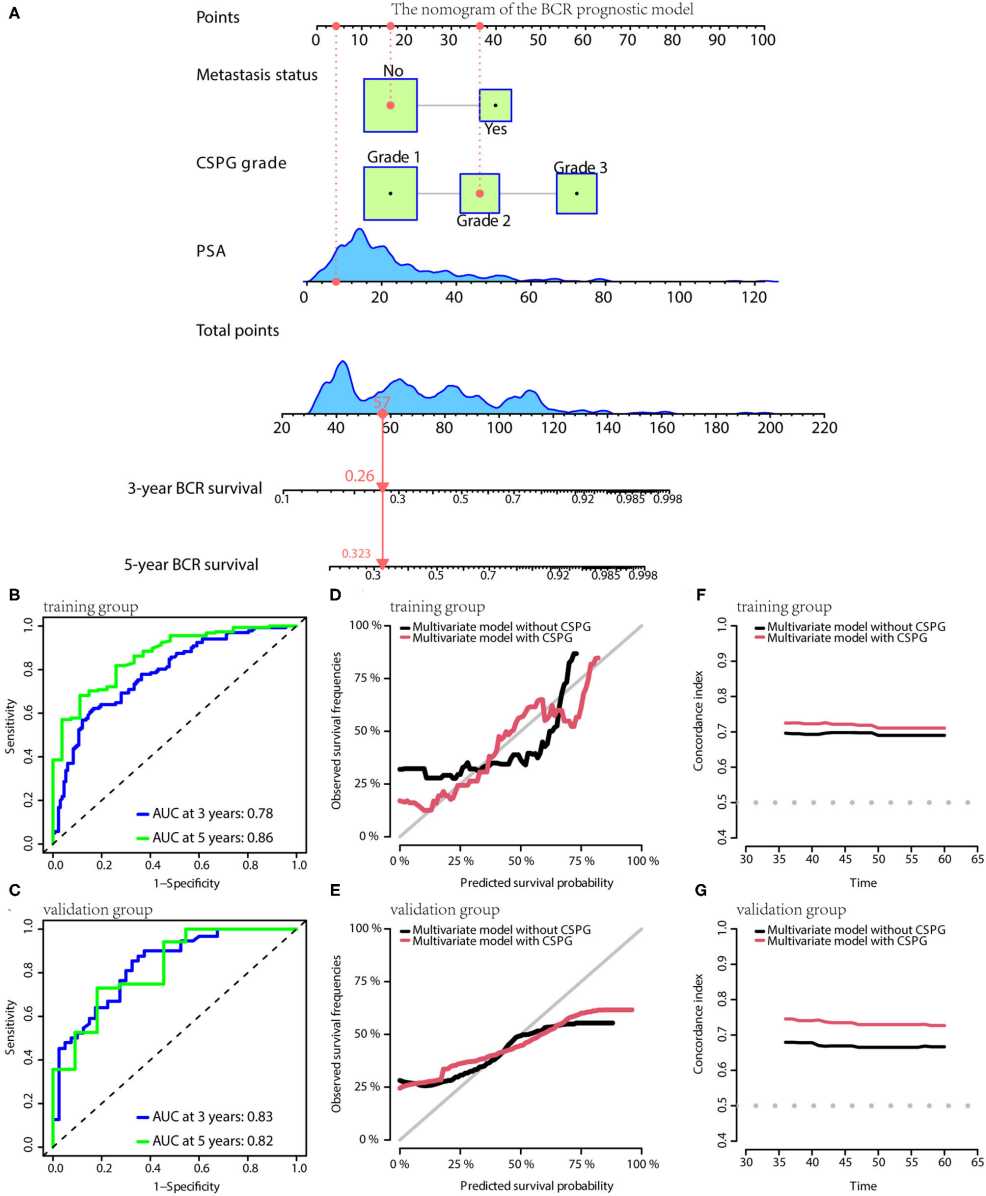
The BCR prognostic model possesses the accuracy and robustness.**(A)** Nomogram of the BCR prognostic model with information of one patient as an example. **(B)** Time-dependent receiver operating characteristic (tdROC) curves show the model has a good predictive accuracy at 3 and 5 years in the training group **(B)** and in the validation group **(C)**. The calibration curves indicate that the model using combined cysteine rich secretory protein 3 (CRISP3) and serine peptidase inhibitor Kazal type 1 (SPINK1) prognostic grade (CSPG) predicts prognosis more accurately in the training group **(D)** and in the validation group **(E)**. The line chart shows the model using CSPG has higher C-index values in the training group **(F)** and in the validation group **(G)**. BCR, biochemical recurrence.

Based on the results of multivariate Cox analyses, three clinical prognostic models using CSPG for BCR, CSS, and OS were developed and validated, respectively. For BCR, the prognostic model consisted of serum PSA, metastasis, and CSPG. The nomogram is shown in Figure 5A. TdROC in the training group showed that the BCR prognostic model had good accuracy (AUC at 3 years: 0.78; AUC at 5 years: 0.86; Figure 5B). Furthermore, tdROC in the validation group revealed that the BCR prognostic model also had good accuracy (AUC at 3 years: 0.83; AUC at 5 years: 0.82; Figure 5C). The calibration curves indicated that the BCR model using CSPG had the potential to more accurately predict prognosis in the training and validation groups (Figures 5D,E). As shown in Figures 5F,G, the BCR prognostic model using CSPG shows higher C-index values in the training and validation groups.

**Figure 6:**
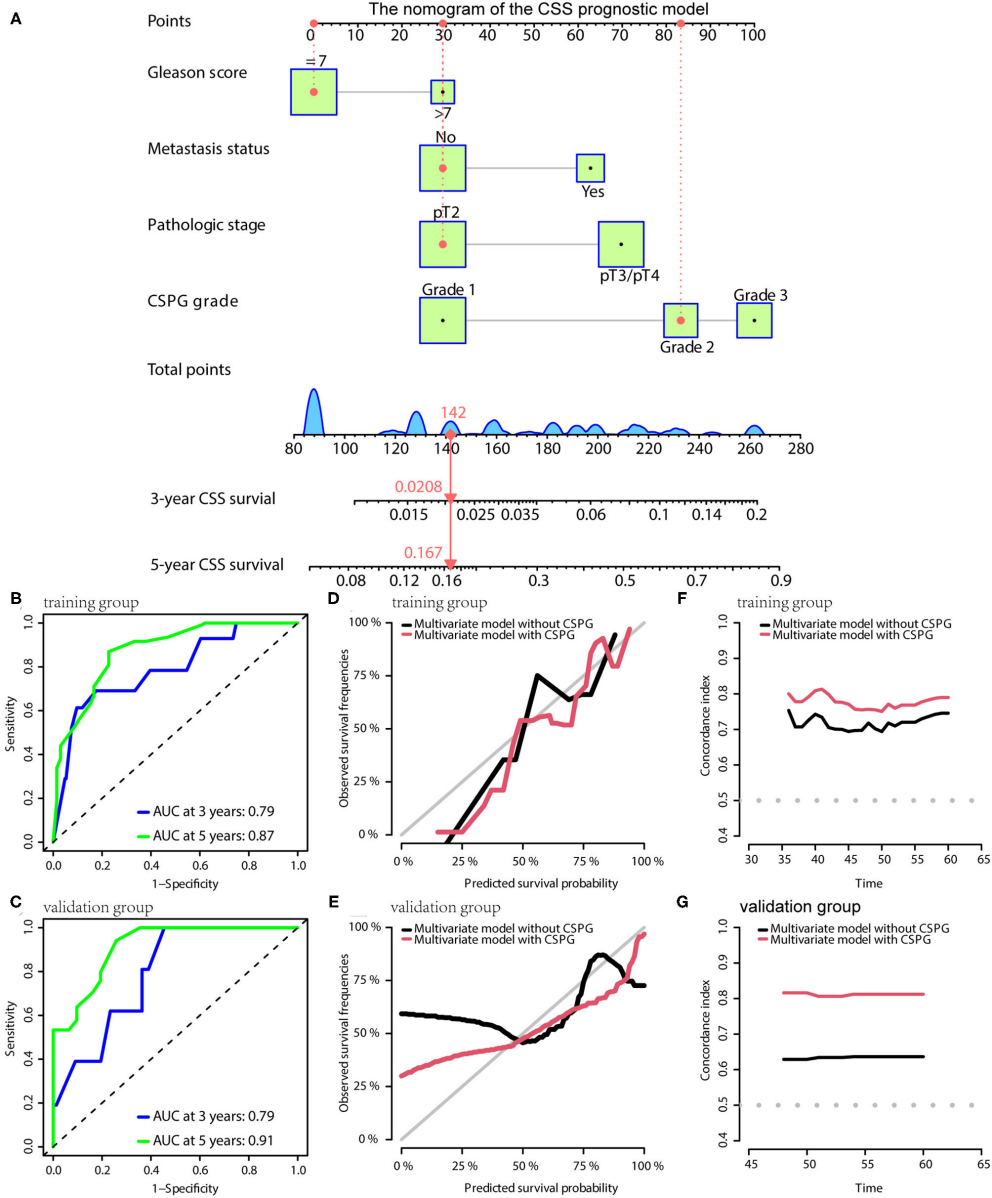
The CSS prognostic model possesses the accuracy and robustness. **(A)** Nomogram of the CSS prognostic model with information of one patient as an example. **(B)** Time-dependent receiver operating characteristic (tdROC) curves show the model has a good predictive accuracy at 3 and 5 years in the training group **(B)** and in the validation group **(C)**. The calibration curves indicate that the model using a combined cysteine rich secretory protein 3 (CRISP3) and serine peptidase inhibitor Kazal type 1 (SPINK1) prognostic grade (CSPG) predicts prognosis more accurately in the training group **(D)** and in the validation group **(E)**. The line chart shows the model using a CSPG has higher C-index values in the training group **(F)** and in the validation group **(G)**. CSS, cancer-specific survival.

For CSS, the prognostic model consisted of Gleason score, metastasis, pathological T stage, and CSPG. The nomogram is given in Figure 6A. TdROC showed that the CSS prognostic model had good accuracy in training (AUC at 3 years: 0.79; AUC at 5 years: 0.87; Figure 6B) and validation groups (AUC at 3 years: 0.79; AUC at 5 years: 0.91; Figure 6C). Similarly, the calibration curves indicated that the CSS prognostic model using CSPG was able to more accurately predict prognosis and showed higher C-index values in the training and validation groups (Figures 6D–G).

**Figure 7:**
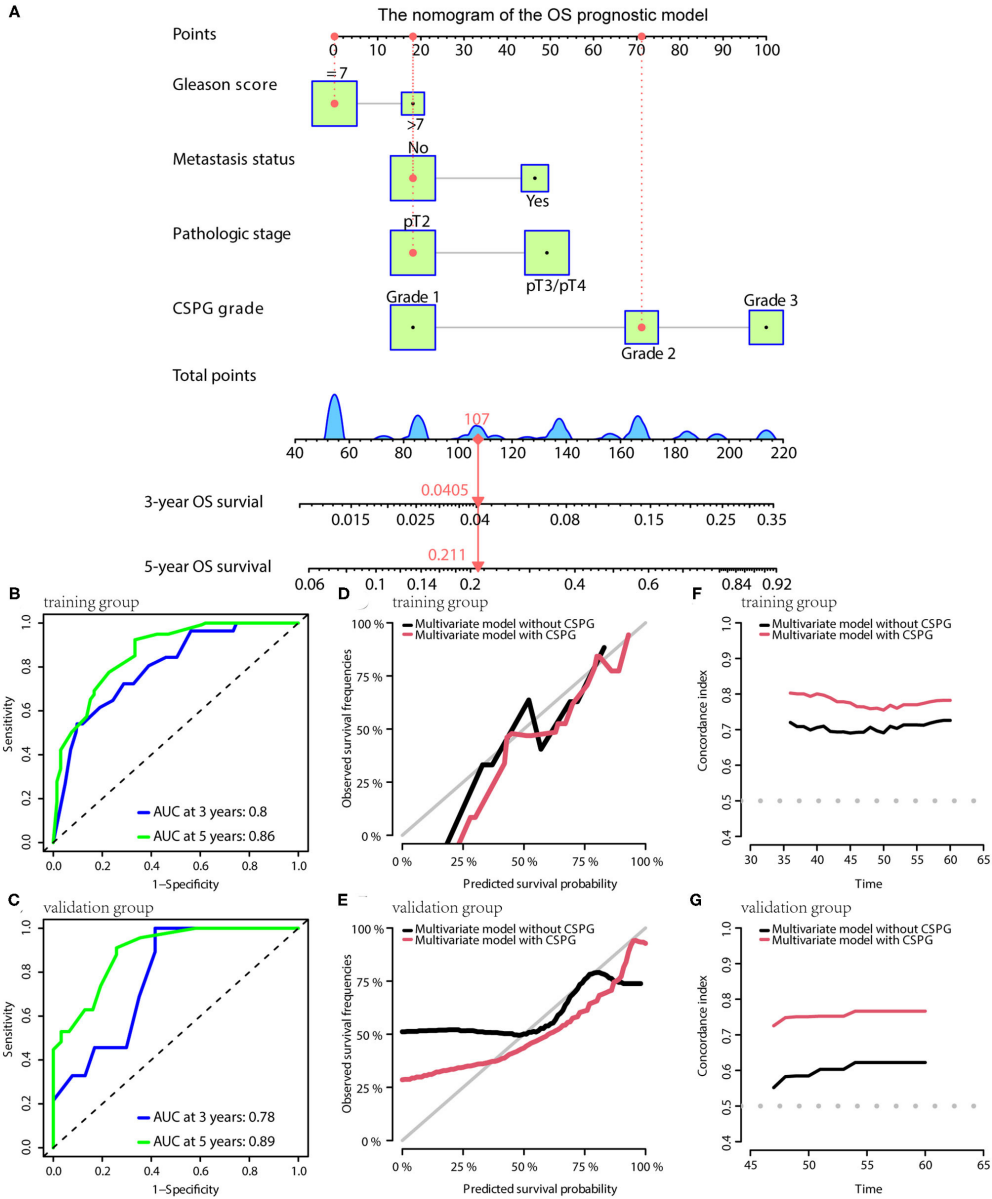
The OS prognostic model possesses the accuracy and robustness. **(A)** Nomogram of the OS prognostic model with information of one patient as an example. Time-dependent receiver operating characteristic (tdROC) curves show the model has a good predictive accuracy at 3 and 5 years in the training group **(B)** and in the validation group **(C)**. The calibration curves indicate that the model using a combined cysteine rich secretory protein 3 (CRISP3) and serine peptidase inhibitor Kazal type 1 (SPINK1) prognostic grade (CSPG) predicts prognosis more accurately in the training group **(D)** and in the validation group **(E)**. The line chart shows the model using a CSPG has higher C-index values in the training group **(F)** and in the validation group **(G)**. OS, overall survival.

For OS, the prognostic model contained Gleason score, metastasis, pathological T stage, and CSPG. The model is visualized *via* the nomogram shown in Figure 7A. Consistently, the OS prognostic model was confirmed to have perfect accuracy *via* tdROC in the training group (AUC at 3 years: 0.80; AUC at 5 years: 0.86; Figure 7B) and validation group (AUC at 3 years: 0.78; AUC at 5 years: 0.89; Figure 7C). In addition, the OS prognostic model using CSPG showed better accuracy and a higher C-index value in the training and validation groups (Figures 7D–G).

In the nomograms of the three models, a patient's information was used as an example and showed it in red line.

## Discussion

As the most common cancer among males, PCa is a major health threat for men worldwide (1, 16). A study investigating the epidemiology of genitourinary tumors over the past 30 years found that PCa remained the major global public health challenge. And this study stated that more proactive intervention strategies, at both the administrative and academic levels, based on the dynamic changes, were needed (17). Furthermore, due to significant PCa heterogeneity, patients present with a variety of outcomes. Therefore, in the context of the strong promotion of precision medicine, it is important to accurately predict patient survival outcome. Prognostic biomarkers have the ability to assist in prognosis judgment and provide the basis for clinicians to make adjuvant treatment decisions after surgery (18). The ability to tailor treatment based on molecular features of disease may potentially reduce the occurrence of unnecessary side effects and ineffective treatments, and thereby reduce both direct and indirect medical costs (19). Pietro Pepe et al. find that prostate cancer gene 3 (PCA3) score in urine improves Prostate Cancer Prevention Trial risk calculator accuracy in PCa diagnosis; moreover, PCA3 score combined with PSA F/T reduce number of unnecessary biopsies (about 20%) (20). The data of another research suggest that urinary PCA3 is more useful as an exclusion tool; moreover, setting a PCA3 cut-off at 20 vs 35, would have avoided 22.9 vs 38.1 % of biopsies while missing 9.4 and 28% diagnosis of PCa (21). As such, it is important to identify and validate new prognostic and predictive molecular biomarkers that may be used to direct cancer treatment.

In this study, CRISP3 and SPINK1 levels in EPS-urine of 496 patients were examined *via* ELISA. These results showed that CRISP3 and SPINK1 levels were increased with the progression of Gleason score, pathological T stage, and metastasis status. Additionally, CRISP3 and SPINK1 had a good predictive effect on prognosis using tdROC curves, and AUC values of CRISP3 and SPINK1 were respectively 0.787 and 0.781. The optimal CRISP3 and SPINK1 cut-off values were respectively 32.1 ng/ml (sensitivity: 73.8%; specificity: 70.0%) and 3,609.2 pg/ml (sensitivity: 61.5%; specificity: 82.5%). CRISP3 and SPINK1 levels were then combined and defined as CSPG. CSPG was used to categorize the prognosis of patients into three grades in survival analyses, grade 1 indicated low risk, grade 2 indicated intermediate risk, and grade 3 indicated high risk. Our results demonstrated that higher CSPG was associated with a worse prognosis. Furthermore, univariate and multivariate Cox analyses indicated that CSPG was an effective independent prognostic variable for BCR, CSS, and OS. It is worthy to note that patients in grade 2 and 3 have a similar CSS. This could be partially explained because grade 2 included PCa patients with elevated CRISP3 or SPINK1, indicating overexpression of either of two biomarkers still predicted poor prognosis. Moreover, the sample size of patients in grade 2 and 3 was relatively limited.

CRISP3 is overexpressed in PCa tissue and higher CRISP3 expression correlates with worse prognosis, which may be caused by increased invasion of cells (8). Noh BJ et al. have revealed that CRISP3 effectively predicts BCR for PCa (9). However, there have been controversial conclusions on the role of SPINK1 in PCa. Richard Flavin et al. have found that SPINK1 expression may not be a predictor of tumor recurrence or prognosis after radical surgery, and SPINK1 and ERG expression are not mutually exclusive patterns (22). ERG and SPINK1 expression may have no significant effect on the metastatic behavior of PCa (23), whereas other studies have suggested a significant association between SPINK1 and progression and prognosis of PCa. SPINK1 overexpression is also reported to be significantly associated with CSS in patients with recurrence after prostatectomy (10). Androgen deprivation causes upregulation of SPINK1, maintaining a neuroendocrine phenotype (24). In addition, previous studies on CRISP3 and SPINK1 were performed in small sample sizes and conducted with invasiveness. In this study, we initially found CRISP3 levels in EPS-urine were significantly correlated with that of SPINK1. To the best of our knowledge, our analysis is the first study to combine these two biomarkers together and demonstrate their predictive value for PCa patients' prognosis, especially based on a relatively large cohort.

Currently, there are many studies on prognostic markers of PCa. Wang et al. have combined albumin and fibrinogen to define a prognostic grade, which predicts prognosis of patients with PCa (25). The degree of heterogeneity within PCa renders the idea of a single holy grail Prostate Cancer Supportive Care marker unlikely. However, a comprehensive panel of selected markers may be the solution. In this study, EPS-urine was used with the advantages of non-invasiveness and convenience. Originally, CRISP3 and SPINK1 were combined into CSPG and it was confirmed that CSPG was effective in stratifying patient risk. In addition, CSPG and conventional clinicopathological indicators were constructed as risk prediction models and comprehensively studied from the perspectives of BCR, CSS, and OS, respectively. Furthermore, the large cohort of 496 patients ensured the reliability of the study conclusions. Our results demonstrated that CSPG and its related models has the potential to evaluate the postoperative prognosis for PCa patients treated with radical prostatectomy, thus to achieve accurate risk stratification and provide intensive monitoring for high risk patients.

Several limitations of our study should be acknowledged. First, the exact mechanism for the observations remain unclear and warrant further investigation. Second, the relationship between CSPG and endocrine therapy or chemotherapy in patients also should be further explored. Third, all these patients were recruited from the same institute, and most cases were from the past 5 years, which was not sufficient for long term follow-up. Further validation of CSPG in a larger, independent, and multicenter cohort with long-term follow-up is still needed.

In conclusion, high CRISP3 and SPINK1 levels in EPS-urine were significantly associated with PCa prognosis. A combined CRISP3 and SPINK1 prognostic grade, defined as CSPG, in EPS-urine accurately predicted prognosis of PCa. Finally, three prognostic models for BCR, CSS, and OS were developed to lay the foundation for further clinical transformations.

## Conclusion

In the present study, we first measured the CRISP3 and SPINK1 expression in the EPS-urine of 496 PCa patients treated with radical prostatectomy. Our results showed that these two proteins in EPS-urine were significant prognosis indicators. Then these two indicators were combined and defined as the combined CRISP3 and SPINK1 prognostic grade (CSPG). Univariate and multivariate Cox analyses indicated CSPG was an effective independent prognostic variable. CSPG and clinicopathologic indicators related to prognosis were also integrated to establish three prognostic models for BCR, CSS and overall survival (OS). Model accuracy was strictly validated in the internal validation group. These findings suggested that CSPG in EPS-urine accurately predicted PCa prognosis.

## Data Availability Statement

The original contributions presented in the study are included in the article/Supplementary Material, further inquiries can be directed to the corresponding author.

## Ethics Statement

The studies involving human participants were reviewed and approved by Ethics Committee of Shengjing Hospital of China Medical University. Written informed consent for participation was not required for this study in accordance with the national legislation and the institutional requirements.

## Author Contributions

LC, EZ, and MZ take responsibility for the integrity of the data and the accuracy of the data analysis in the study and were responsible for the design and conception of the research project. LC, EZ, JG, ZC, JY, WL, BY, YS, and MZ contributed to data acquisition or data analysis and data cleaning. LC, EZ, JG, and MZ participated in the drafting of the manuscript and the rigorous modification of the manuscript to clearly convey the research contents. All authors are responsible for the authenticity and reliability of this study and have no objection to the final submitted manuscript.

## Funding

This research was supported by the National Natural Science Foundation of China (Nos. 81802540 and 82173372), the Natural Science Foundation of Liaoning Province (No. 20180550985), the Shenyang Science and Technology Program for Young Innovative Talents (No. RC190386), and the 345 Talent Project of Shengjing Hospital of China Medical University (30 project) for sample collection, data collection, analysis, and interpretation.

## Conflict of Interest

The authors declare that the research was conducted in the absence of any commercial or financial relationships that could be construed as a potential conflict of interest.

## Publisher's Note

All claims expressed in this article are solely those of the authors and do not necessarily represent those of their affiliated organizations, or those of the publisher, the editors and the reviewers. Any product that may be evaluated in this article, or claim that may be made by its manufacturer, is not guaranteed or endorsed by the publisher.
