# Supplementary material for: A Combined CRISP3 and SPINK1 Prognostic Grade in EPS-Urine and Establishment of Models to Predict Prognosis of Patients With Prostate Cancer
Source: Front Med (Lausanne). 2022 Feb 17;9:832415. doi: 10.3389/fmed.2022.832415 (PMC8891445; doi:10.3389/fmed.2022.832415)

Supplementary Material

Supplementary Material

1 Supplementary Figure 1

Scatter diagram indicated that the levels of SPINK1 and CRISP3 in EPS-urine were significantly associated. The Pearson correlation coefficients in all patients, and patients with different risk were displayed also (*** means P < 0.001).

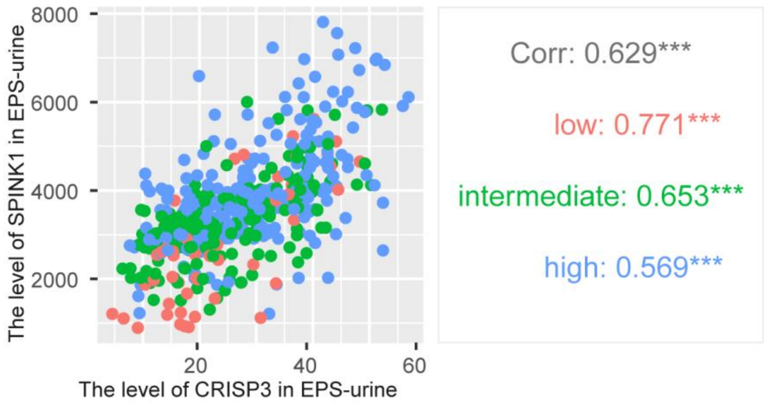

Supplement: Supplementary file 1 [file Data_Sheet_1.PDF]
